# CD39 Expression Identifies Terminally Exhausted CD8^+^ T Cells

**DOI:** 10.1371/journal.ppat.1005177

**Published:** 2015-10-20

**Authors:** Prakash K. Gupta, Jernej Godec, David Wolski, Emily Adland, Kathleen Yates, Kristen E. Pauken, Cormac Cosgrove, Carola Ledderose, Wolfgang G. Junger, Simon C. Robson, E. John Wherry, Galit Alter, Philip J. R. Goulder, Paul Klenerman, Arlene H. Sharpe, Georg M. Lauer, W. Nicholas Haining

**Affiliations:** 1 Department of Pediatric Oncology, Dana-Farber Cancer Institute, Boston, Massachusetts, United States of America; 2 Peter Medawar Building for Pathogen Research, University of Oxford, Oxford, United Kingdom; 3 Department of Microbiology and Immunobiology and Evergrande Center for Immunologic Diseases, Harvard Medical School and Brigham and Women’s Hospital, Boston, Massachusetts, United States of America; 4 Gastrointestinal Unit, Massachusetts General Hospital and Harvard Medical School, Massachusetts, United States of America; 5 Department of Microbiology and Institute for Immunology, University of Pennsylvania Perelman School Medicine, Philadelphia, Pennsylvania, United States of America; 6 Ragon Institute of Massachusetts General Hospital, Harvard University and Massachusetts Institute of Technology, Cambridge, Massachusetts, United States of America; 7 Department of Surgery, Beth Israel Deaconess Medical Center, Harvard Medical School, Boston, Massachusetts, United States of America; 8 Division of Gastroenterology, Department of Medicine, Beth Israel Deaconess Medical Center, Harvard University, Boston, Massachusetts, United States of America; 9 Broad Institute of MIT and Harvard, Cambridge, Massachusetts, United States of America; 10 Division of Hematology/Oncology, Children's Hospital, Harvard Medical School, Boston, Massachusetts, United States of America; Vaccine Research Center, UNITED STATES

## Abstract

Exhausted T cells express multiple co-inhibitory molecules that impair their function and limit immunity to chronic viral infection. Defining novel markers of exhaustion is important both for identifying and potentially reversing T cell exhaustion. Herein, we show that the ectonucleotidse CD39 is a marker of exhausted CD8^+^ T cells. CD8^+^ T cells specific for HCV or HIV express high levels of CD39, but those specific for EBV and CMV do not. CD39 expressed by CD8^+^ T cells in chronic infection is enzymatically active, co-expressed with PD-1, marks cells with a transcriptional signature of T cell exhaustion and correlates with viral load in HIV and HCV. In the mouse model of chronic Lymphocytic Choriomeningitis Virus infection, virus-specific CD8^+^ T cells contain a population of CD39^high^ CD8^+^ T cells that is absent in functional memory cells elicited by acute infection. This CD39^high^ CD8^+^ T cell population is enriched for cells with the phenotypic and functional profile of terminal exhaustion. These findings provide a new marker of T cell exhaustion, and implicate the purinergic pathway in the regulation of T cell exhaustion.

## Author Summary

Chronic viral infection induces an acquired state of T cell dysfunction known as exhaustion. Discovering surface markers of exhausted T cells is important for both to identify exhausted T cells as well as to develop potential therapies. We report that the ectonucleotidase CD39 is expressed by T cells specific for chronic viral infections in humans and a mouse model, but is rare in T cells following clearance of acute infections. In the mouse model of chronic viral infection, CD39 demarcates a subpopulation of dysfunctional, exhausted CD8^+^ T cells with the phenotype of irreversible exhaustion. CD39 expression therefore identifies terminal CD8^+^ T cell exhaustion in mice and humans, and implicates the purinergic pathway in the regulation of exhaustion.

## Introduction

In acute infections, antigen-specific T cells differentiate into activated effector cells and then into memory T cells which rapidly gain effector functions and re-expand on subsequent encounter with the same pathogen [1]. In contrast, during chronic infections, pathogen-specific T cells gradually lose effector functions, fail to expand, and can eventually become physically deleted [2]. These traits are collectively termed T cell exhaustion, and have been described both in animal models of chronic viral infection as well as in human infections with hepatitis C virus (HCV) and human immunodeficiency virus (HIV) [2–4]. Identifying reversible mechanisms of T cell exhaustion is therefore a major goal in medicine.

Prolonged or high-level expression of multiple inhibitory receptors such as PD-1, Lag3, and CD244 (2B4) is a cardinal feature of exhausted T cells in both animal models and human disease [5–7]. Expression of PD-1 appears to be a particularly important feature of exhausted CD8^+^ T cells, as the majority of exhausted cells in mouse models of chronic infection express this receptor, and blockade of the PD-1:PD-L1 axis can restore the function of exhausted CD8^+^ T cells in humans and mouse models [2,6]. However, in humans, many inhibitory receptors also can be expressed by a large fraction of fully functional memory CD8^+^ T cells. PD-1, for instance, can be expressed by up to 60% of memory CD8^+^ T cells in healthy individuals, making it challenging to use PD-1 to identify exhausted CD8^+^ T cells in humans, particularly when the antigen-specificity of potentially exhausted CD8^+^ T cells is not known [8].

Studies in mice and humans suggest that exhausted CD8^+^ T cells are not a homogeneous population, but instead include at least two subpopulations of T cells that differentially express the transcription factors T-bet and Eomesodermin (Eomes) [9–11]. T-bet^high^ CD8^+^ T cells represent a progenitor subset with proliferative potential that give rise to Eomes^high^ CD8^+^ T cells, which are terminally differentiated and can no longer proliferate in response to antigen or be rescued by PD-1 blockade [9,12]. Both populations express PD-1, but Eomes^high^ exhausted cells express the highest levels of PD-1. However, no specific cell-surface markers of this terminally differentiated population of exhausted cells have thus far been identified.

CD39 (*ENTPD1*) is an ectonucleotidase originally identified as an activation marker on human lymphocytes and as the vascular ecto-ADPase [13], but has subsequently been shown to be a hallmark feature of regulatory T cells [14–16]. CD39 hydrolyzes extracellular ATP and ADP into adenosine monophosphate, which is then processed into adenosine by CD73, an ecto-5'-nucleotidase [17]. Adenosine is a potent immunoregulator that binds to A2A receptors expressed by lymphocytes causing accumulation of intracellular cAMP, preventing T cell activation and NK cytotoxicity [18–20]. Loss of CD39 in Tregs markedly impairs their ability to suppress T cell activation, suggesting that the juxtacrine activity of CD39 serves to negatively regulate T cell function [15]. However, blood CD8^+^ T cells have generally been reported to be CD39^–^ [14,21–23], and the expression of this marker on exhausted T cells has not been examined.

In this study, we demonstrate that, in contrast to CD8^+^ T cells from healthy donors, antigen-specific CD8^+^ T cells responding to chronic viral infection in humans and a mouse model express high levels of biochemically active CD39. CD39^+^ CD8^+^ T cells co-express PD-1 and are enriched for a gene signature of T cell exhaustion. In the mouse model of chronic LCMV infection, high levels of CD39 expression demarcate terminally differentiated virus-specific CD8^+^ T cells within the pool of exhausted CD8^+^ T cells. Thus, CD39 provides a specific, pathological marker of exhausted CD8^+^ T cells in chronic viral infection in humans and mouse models of chronic viral infection.

## Results

### CD39 is expressed by CD8^+^ T cells responding to chronic infection

**Fig 1:**
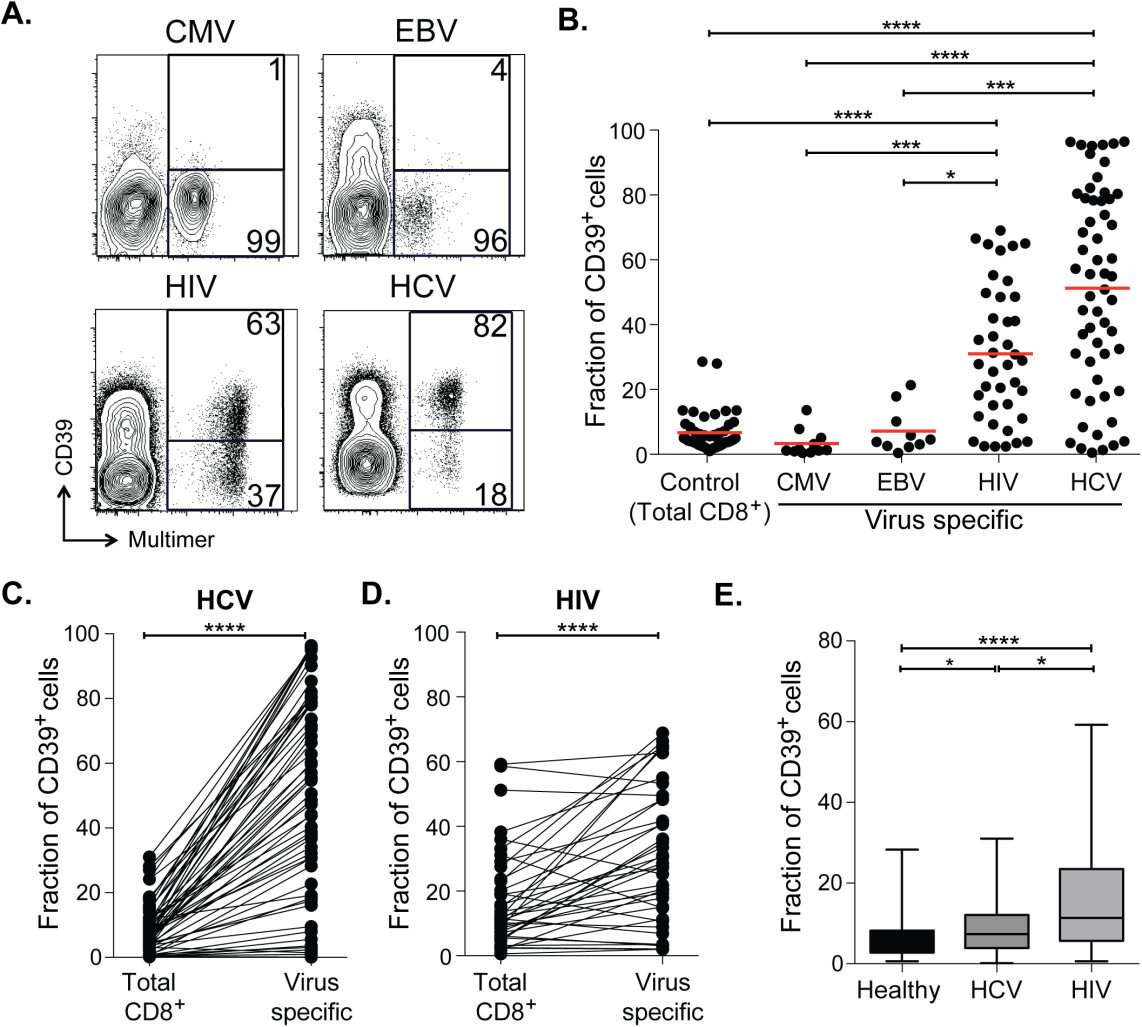
CD39 is highly expressed by virus-specific CD8^+^ T cells in chronic viral infection. **(A)** Expression of CD39 by virus-specific CD8^+^ T cells. Plots are gated on CD8^+^. **(B)** Fraction of total or antigen-specific CD8^+^ T cells expressing CD39. **(C, D)** Comparison of CD39 expression by total CD8^+^ T cells with virus-specific CD8^+^ T cells from patients with HCV (C) and HIV (D) infections. **(E)** Fraction of total CD8^+^ T cells expressing CD39 in healthy, HIV or HCV infected donors. Error bars represent SEM. Statistical significance was assessed by Kruskal-Wallis test (B, E), or Wilcoxon test (C, D). *P <0.05, ***P <0.001, ****P <0.0001.

We surveyed the expression of CD39 by CD8^+^ T cells from healthy adult subjects without chronic viral infection. Consistent with previous reports we found that only a small fraction (mean 6%) of CD8^+^ T cells in healthy individuals expressed CD39 (Fig 1A and 1B) [14,21–23]. This small population of CD39^+^ CD8^+^ T cells in healthy donors was primarily found in the central and effector memory compartments while virtually no naive CD8^+^ T cells expressed CD39 (S1 Fig). We next focused on CD39 expression by antigen-specific CD8^+^ T cells specific for latent viruses in healthy subjects and found that only a very small fraction of CMV- or EBV-specific CD8^+^ T cells expressed CD39 (Fig 1A and 1B) (mean 3% and 7% respectively).

We next measured CD39 expression by T cells specific for the chronic viral pathogens HCV and HIV. We measured CD39 expression in 57 subjects with acute HCV infections (23 with acute resolving infection and 34 with chronically evolving infection), and in 40 subjects with HIV infection (28 chronic progressors and 12 controllers; clinical characteristics of the subjects are summarized in S1 Table). We found a mean of 51% of HCV-specific CD8^+^ T cells and 31% of HIV-specific CD8^+^ T cells expressed CD39, a number significantly higher than CD8^+^ T cells specific for EBV or CMV, or in total CD8^+^ T cell populations from healthy individuals (Fig 1A and 1B). A slightly greater fraction of virus-specific CD8^+^ T cells from HCV-infected subjects expressed CD39 than did those from HIV-infected subjects.

In subjects with chronic infection, the frequency of CD39-expressing cells in the virus-specific (tetramer^+^) CD8^+^ T cell population was significantly higher than in the total CD8^+^ T cell population (Fig 1C and 1D). However the fraction of total CD8^+^ T cells expressing CD39 in the CD8^+^ T cell compartment of individuals with HCV or HIV infection was slightly increased compared to healthy controls (Fig 1E), consistent with the presence of other, unmeasured virus-specific CD8^+^ T cells that were also CD39^+^ in the tetramer^−^fraction of CD8^+^ T cells. Thus CD39 is expressed infrequently by CD8^+^ T cells in healthy donors, but marks a large fraction of pathogen-specific cells CD8^+^ T cells in patients with chronic infection.

### CD39 expressed by CD8^+^ T cells hydrolyzes ATP

**Fig 2:**
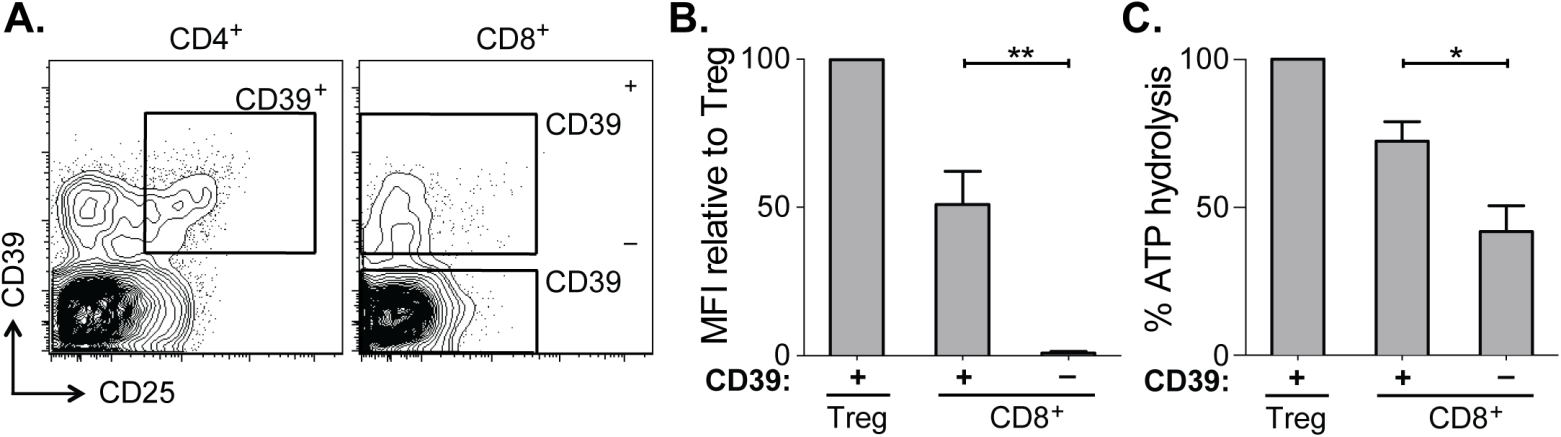
CD39 expressed by CD8^+^ T cells in HCV infection is enzymatically active. **(A)** Flow cytometry sorting gates of CD39^+^ and CD39^–^ CD8^+^ T cells and CD39^+^ CD25^+^ CD4^+^ Tregs used for rpHPLC analysis of CD39 activity. **(B)** Summary of CD39 expression level relative to Tregs in the same subjects. **(C)** ATP hydrolysis by CD8^+^ T cell populations relative to Tregs. Data represent 6 patients with chronic HCV infection. Error bars represent SEM. Statistical significance was assessed by paired Student’s t-test (B, C). *P <0.05, **P <0.01.

CD39 expressed by regulatory T cells catalyzes the hydrolysis of ADP to 5’-AMP [14–16] but its enzymatic activity can be regulated by a range of post-transcriptional mechanisms [PMID. We therefore tested CD39 expressed by CD8^+^ T cells from patients infected with chronic HCV was functional using ATP hydrolysis as a surrogate marker of CD39 activity [24–26]. We sorted CD39^–^ and CD39^+^ CD8^+^ T cells from six HCV-infected individuals (four with chronic infection and two with resolved infection) and incubated equal numbers of cells in the presence of extracellular ATP (eATP). Remaining levels of eATP were measured in the supernatant by HPLC. As a control, we assessed ATP hydrolysis by CD4^+^ CD25^+^ CD39^+^ regulatory T cells (Tregs) sorted from the same individuals (Fig 2A).

Within the CD39^+^ CD8^+^ T cell population the level of CD39 expression was lower than in Tregs (Fig 2B). Consistent with reduced CD39 expression relative to Tregs, ATP hydrolysis by CD39^+^ CD8^+^ T cells was less than that by Tregs (Fig 2C). However ATP hydrolysis by CD39^+^ CD8^+^ T cells was significantly greater than that of CD39^–^ cells (Fig 2C). Thus CD39 expressed by CD8^+^ T cells in HCV infection is enzymatically active and capable of hydrolyzing ATP.

### CD39 is co-expressed with PD-1 on virus-specific CD8^+^ T cells and correlates with viral load in both HCV and HIV infection

**Fig 3:**
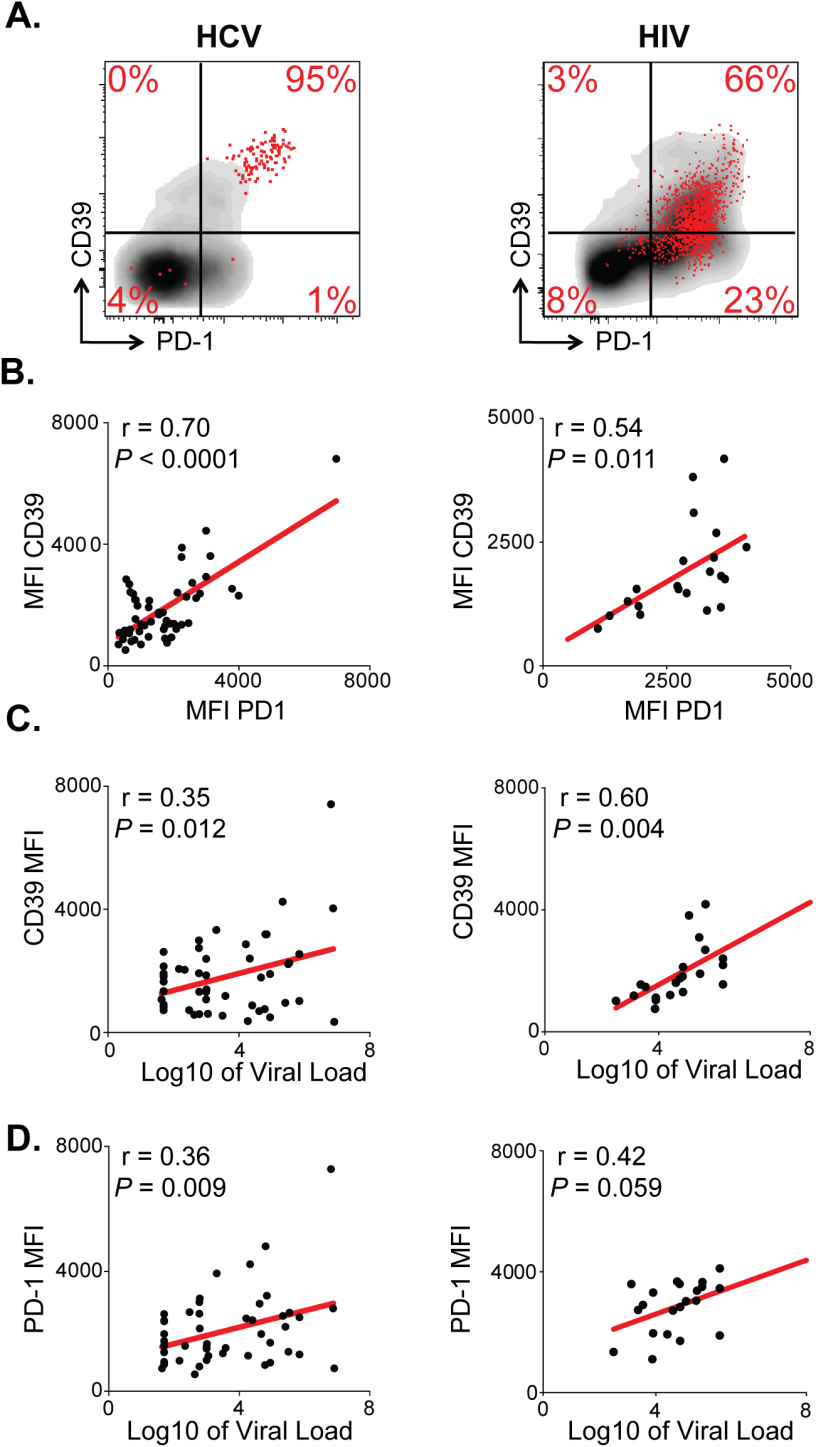
CD39 expression correlates with PD-1 expression and viral load in chronic viral infection. **(A)** CD39 and PD-1 expression in chronic HCV (left) or HIV infection (right). Representative plots demonstrate total (gray) and virus-specific (red) CD8^+^ T cells. **(B)** Correlation between CD39 and PD-1 expression by HCV- (left) and HIV-specific (right) CD8^+^ T cells. **(C)** Correlation between CD39 expression by virus-specific CD8^+^ T cells and viral load count in HCV (left) or HIV (right) infection. **(D)** Correlation between PD-1 expression by virus-specific CD8^+^ T cells and viral load in HCV (left) or HIV (right) infection. Correlation was assessed by Pearson correlation coefficient (B, C, D). MFI; mean fluorescence intensity.

CD8^+^ T cells specific for chronic viruses such as HCV and HIV express increased levels of PD-1 [3,27]. We therefore examined the relationship between CD39 and PD-1 expression by virus-specific CD8^+^ T cells in 54 patients with HCV (23 chronically infected and 31 resolvers) and 40 patients infected with HIV (28 chronic progressors, 7 viremic controllers and 5 elite controllers). In both diseases we found a significant association between the level of expression (mean fluorescence intensity, MFI) of CD39 and PD-1 on antigen-specific CD8^+^ T cells in subjects with HCV and with HIV (r = 0.70, P <0.0001 and r = 0.54, P<0.05, respectively) (Fig 3A and 3B).

We next examined the relationship between CD39 and PD-1 expression and viral load in HCV and HIV infection. We found that in both the HCV and HIV infection there was a modest but significant correlation between viral load and the level of CD39 expression on virus-specific CD8^+^ T cells measured by MFI (Fig 3C). The fraction of CD39^+^, virus-specific CD8^+^ T cells was significantly higher in HIV progressors compared with those from HIV controllers (S2 Fig). A similar, but non-significant, trend was seen comparing CD39 expression in HCV-specific CD8^+^ T cells in patients with chronic versus resolved disease. However, in HCV, a significantly higher fraction of virus-specific CD8^+^ T cells co-expressed both CD39 and PD-1 in patients with chronic versus resolved disease (S2 Fig). Consistent with these findings, there was a significant correlation between viral load and the fraction of virus-specific CD8^+^ T cells that were CD39^+^ PD-1^+^ double positive in both HCV and HIV infection (S2 Fig). PD-1 expression was also modestly correlated with the viral load in HCV and in HIV-infected patients (Fig 3D) [3,27]. Thus CD39 expression by virus-specific CD8^+^ T cells is greatest in setting of high antigen burden.

### Transcriptional analysis of CD39^+^ CD8^+^ T cells in HCV infection

In order to characterize more broadly the phenotype of CD39^+^ CD8^+^ T cells from individuals with chronic infection, we compared the global gene expression profiles of sorted CD39^+^ and CD39^–^ CD8^+^ T cells from 8 HCV-infected subjects (3 with acute resolving infection and 5 with chronically evolving infection; S4 Table). Limited numbers of cells precluded the comparison of CD39^+^ and CD39^–^ CD8^+^ T cells within HCV-specific cells, leading us to focus on the total CD8^+^ population of antigen-experienced CD8^+^ T cells (S4 Table). Because naive CD8^+^ T cells express little CD39 (S1 Fig), we excluded this population from the sorted cells (S3 Fig) to enable direct comparison of antigen-experienced CD39^+^ and CD39^–^ CD8^+^ T cells.

**Fig 4:**
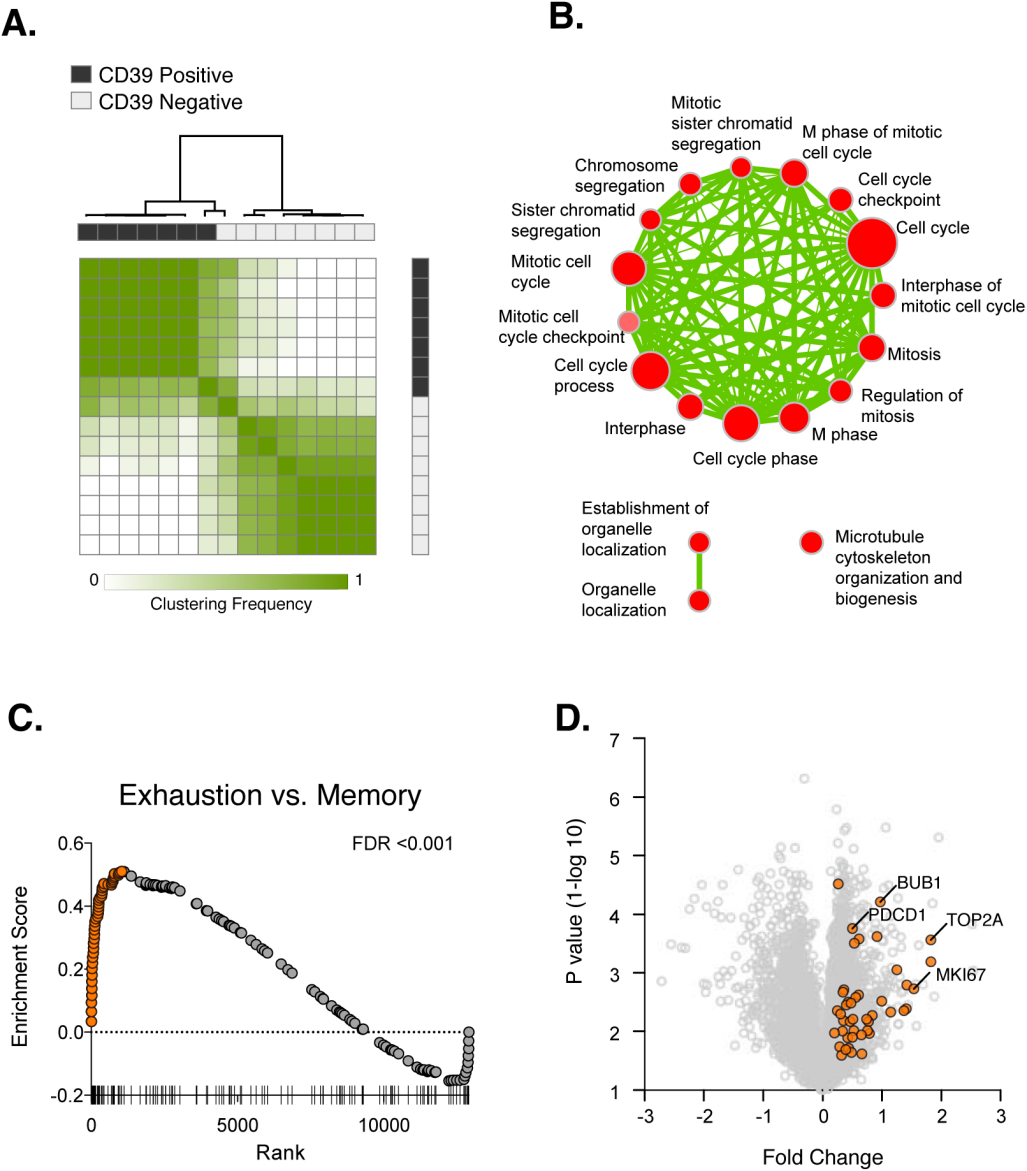
Transcriptional analysis of CD39^+^ and CD39^–^ CD8^+^ T cells in HCV infection. **(A)** Consensus hierarchical clustering of expression profiles from CD39^+^ (black) and CD39^–^ (grey) CD8^+^ T cells from 8 HCV infected patients. Clustering is based on the top 10% of genes by variance across the dataset. Sample similarity (1-Pearson correlation coefficient) is annotated with color from low (white) to high (green). **(B)** Gene set enrichment map displaying Gene Ontology gene sets enriched (FDR < 0.1) in CD39^+^ CD8^+^ T cells from (A). Nodes (in red) are sized in proportion to gene set size; connecting line thickness represents extent of gene member overlap between gene sets. **(C)** Gene set enrichment analysis of a signature of 200 genes up-regulated in exhausted CD8^+^ T cells from the mouse model of chronic viral infection versus acute infection (day 30 post infection) in the ranked list of genes differentially expressed by CD39^+^ vs. CD39^–^ CD8^+^ T cells. Leading edge genes are indicated by orange symbols. **(D)** Volcano plot of all genes (grey) or exhausted leading edge genes (orange) from (C).

We first used unbiased clustering approaches to identify whether CD39^+^ and CD39^–^ CD8^+^ T cells showed distinct patterns of gene expression. Analysis of gene expression profiles using consensus hierarchical clustering (Fig 4A) showed two distinct clusters of samples that corresponded almost exactly to CD39^+^ and CD39^–^ populations, suggesting that that in both acute and chronic infection, CD39 expression demarcates two types of CD8^+^ T cells with markedly different patterns of gene expression. Supervised analysis of differential gene expression identified 619 genes differentially expressed (FDR<0.15) between CD39^+^ and CD39^–^ CD8^+^ T cells (S4 Table). Inspection of the list of differentially expressed genes revealed many with known roles in CD8^+^ T cell biology including increased expression of the inhibitory receptors PD-1 and CTLA-4 in CD39^+^ CD8^+^ T cells.

To identify biological processes that were differentially active in CD39^+^ vs. CD39^–^ cells, we performed gene set enrichment analysis using the Gene Ontology collection of gene sets [28]. We found no significant enrichment of GO terms in the CD39^–^ CD8^+^ subset. In contrast, 21 gene sets significantly enriched (FDR<0.1) in CD39^+^ population, almost all of which were related to mitosis and cell-cycle associated genes or cytoskeleton organization (Fig 4B). This suggests that CD39^+^ CD8^+^ T cells in chronic viral infection show coordinate up-regulation of genes related to proliferation.

The expression of CD39 by CD8^+^ T cells in chronic but not acute/latent infection, suggests that it may be a marker of T cell exhaustion. We therefore tested whether the profile of CD39^+^ CD8^+^ T cells was enriched for genes expressed by exhausted CD8^+^ cells. Previous studies of gene expression in CD8^+^ T cells in the mouse model of chronic viral infection with the Clone 13 strain of LCMV have identified signatures of T cell exhaustion that are also enriched in exhausted CD8^+^ T cells in humans [29–31]. We therefore curated a signature of 200 genes up-regulated by exhausted CD8^+^ T cells responding to chronic infection relative to functional memory CD8^+^ T cells generated by acute infection (LCMV Armstrong strain). We found that the exhausted CD8^+^ T cell signature from LCMV model was significantly enriched in CD39^+^ vs. CD39^–^ CD8^+^ T cells in subjects with HCV infection (Fig 4C). We focused on the “leading edge” genes contributing most to the enrichment [32], which correspond to genes up-regulated both in the mouse exhausted signature and in the human CD39^+^ profile. As expected, the leading edge genes included PD-1 (*PDCD1*), a feature of both human CD39^+^ CD8^+^ T cells and of exhausted CD8^+^ T cells in the mouse model (Fig 4D). In addition we found that up-regulation of many genes associated with proliferation including *BUB1*, *TOP2A* and *MKI67* was common to mouse exhausted CD8^+^ T cells and human CD39^+^ CD8^+^ T cells. Thus CD39^+^ CD8^+^ T cells in HCV infection and exhausted CD8^+^ T cells in a mouse model of chronic infection share transcriptional features that include genes related to proliferation.

### CD39 is increased in exhausted CD8^+^ T cells in the mouse model of chronic LCMV infection

Because the mouse signature of CD8^+^ T cell exhaustion was significantly enriched in the transcriptional profile of CD39^+^ CD8^+^ T cells in HCV-infected patients, we next asked if CD39 was up-regulated by CD8^+^ T cells in the mouse model of chronic viral infection. To address this question we compared two well-described mouse models of viral infection using two strains of LCMV: LCMV Armstrong that causes an acute infection that is resolved in up to 8 days; and LCMV Clone 13 that persists in mice for up to 3 months and leads to T cell exhaustion [5,6].

**Fig 5:**
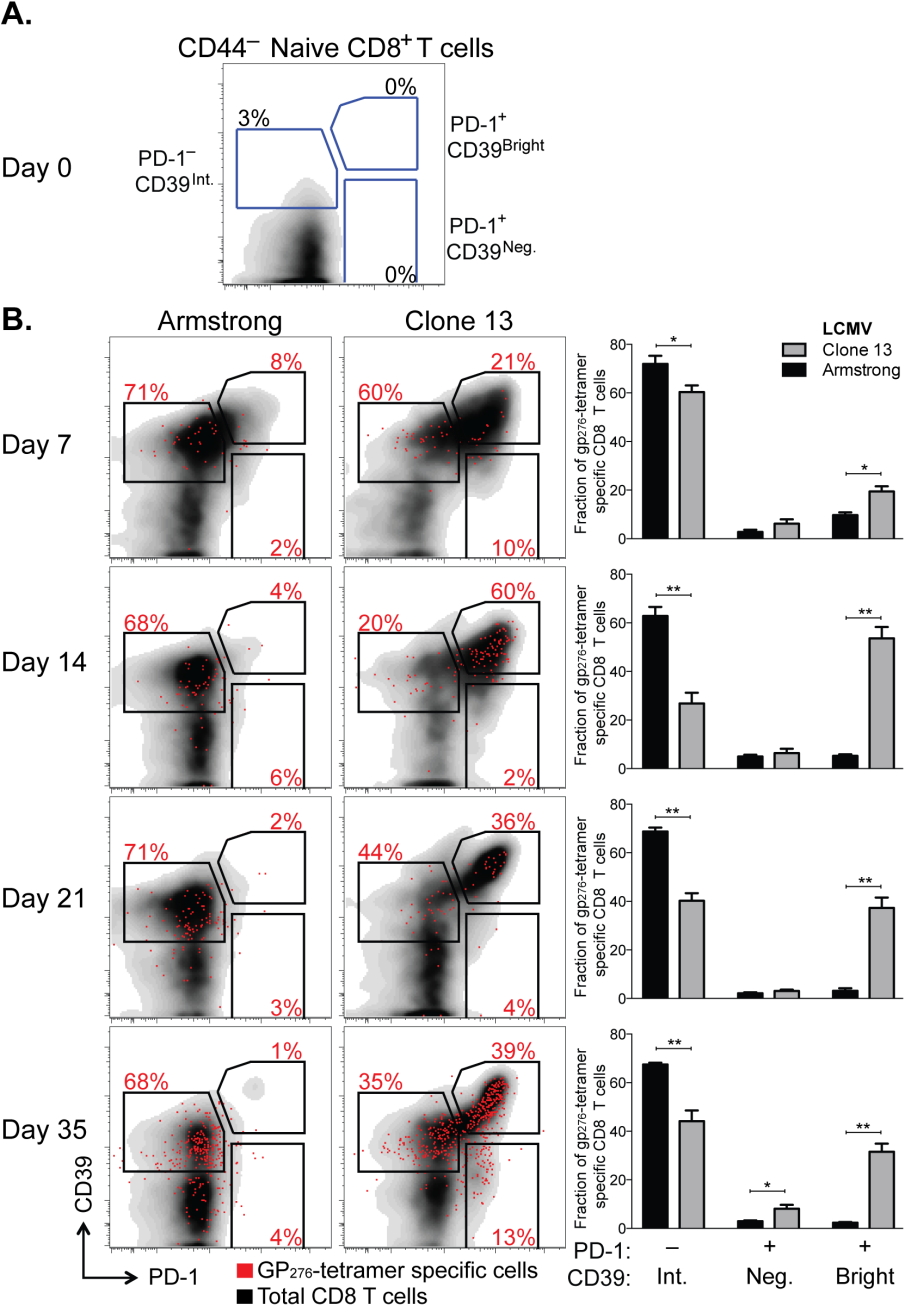
CD39 is highly up-regulated by exhausted CD8^+^ T cells in a mouse model of chronic infection. (**A, B**) Expression of CD39 and PD-1 by CD44^–^ naive mouse CD8^+^ T cells (A) and in CD8^+^ T cells at indicated times following LCMV Armstrong (acute) or Clone 13 (chronic) infection (B). Representative plots show total (black) and H-2Db GP_276-286_ tetramer-specific CD8^+^ T cells (red). Summary of results in 5 mice per group is shown in bar-graphs on the right. Statistical significance was assessed with Mann-Whitney test. *P < 0.5, **P < 0.01.

We measured CD39 expression and compared it to PD-1 expression in CD8^+^ T cells responding to each infection. While naive CD8^+^ T cells expressed neither CD39 nor PD-1 (Fig 5A), both were rapidly and coordinately up-regulated by antigen-experienced cells following either infection (day 7 post infection [d7 p.i.], Fig 5B). However, in acute infection, the fraction of CD39 bright PD-1^+^ population decreased with time. In contrast, high expression of CD39 and PD-1 was maintained in Clone 13 infection. The accumulation of CD39 bright PD-1^+^ cells among the total CD8^+^ population was most apparent in the H-2D^b^ GP_276-286_ tetramer-specific CD8^+^ T cells (Fig 5B).

Thus after chronic viral infection, antigen-specific CD8^+^ T cells can be identified by high expression of both CD39 and PD-1. This difference in expression of both markers between chronic and acute infection is noticeable as early as d7 p.i. but becomes more pronounced with time after infection.

### CD39 expression correlates with a terminally exhausted phenotype in virus-specific CD8^+^ T cells in chronic infection

**Fig 6:**
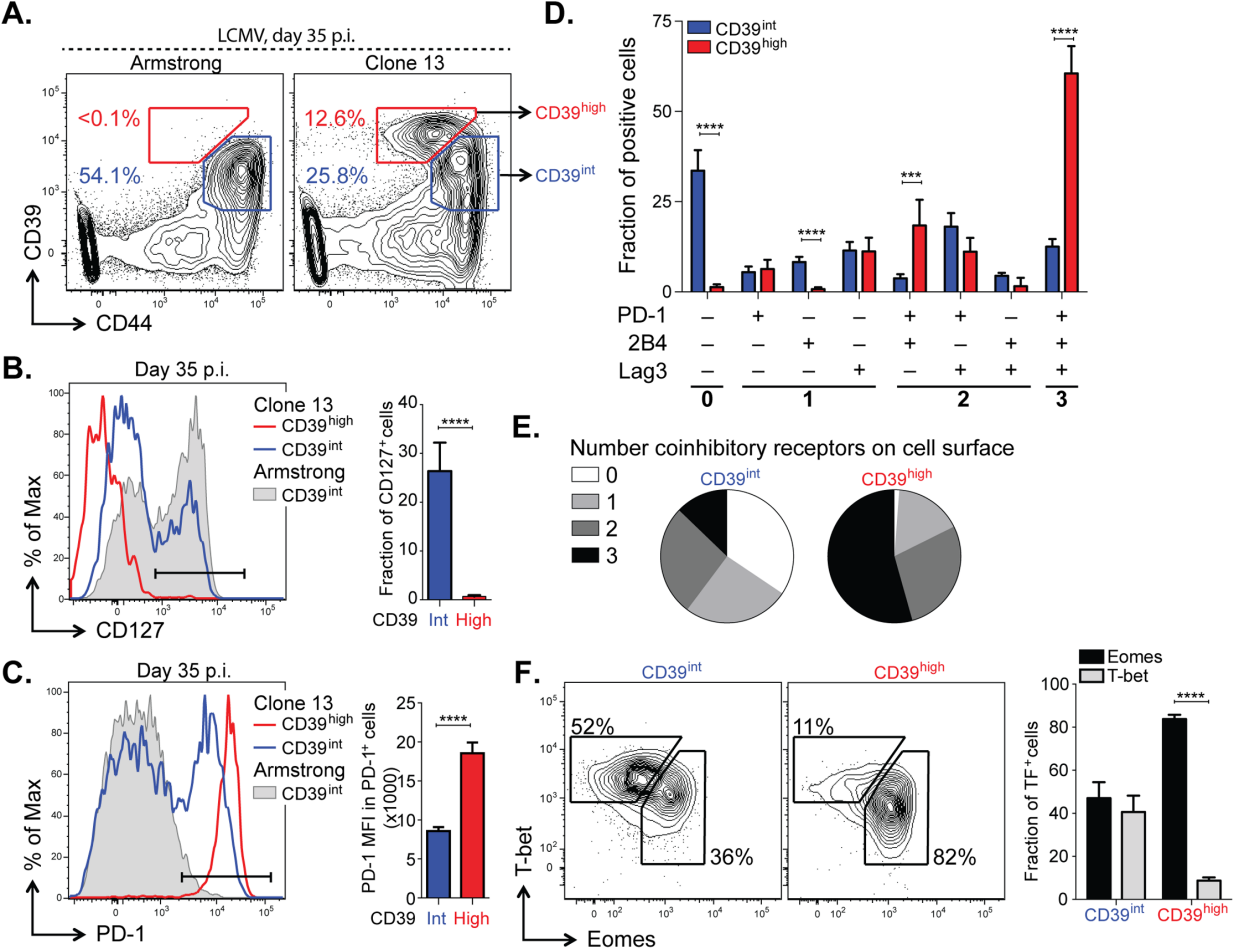
CD39 identifies terminally exhausted CD8^+^ T cells in mice with chronic LCMV infection. (**A**) Expression of CD39 and CD44^+^ by mouse CD8^+^ T cells 30–35 days following LCMV Armstrong (left) or Clone 13 (right) infection. (**B**, **C**) Representative histograms (left) of CD127 (B) and PD-1 (C) expression by CD39^high^ and CD39^int^ CD8^+^ T cells from Clone 13 (red and blue, respectively) and CD39^int^ from Armstrong (filled gray) infected mice on d35 p.i. (left). Fraction of CD127^+^ (B) and MFI of PD-1 in PD-1^+^ cells (C) is shown on the right. Results are from 5 mice. (**D**) Fraction of CD39^high^ and CD39^int^ CD44^+^ CD8^+^ T cells expressing different combinations of co-inhibitory receptors PD-1, 2B4, and Lag3. (**E**) Average number of co-inhibitory receptors expressed by CD39^int^ (left) or CD39^high^ (right) CD8^+^ T cells at d35 p.i. following LCMV Clone 13 infection. (**F**) Representative plots of T-bet and Eomes expression in CD39^int^ (left) and CD39^high^ (right) cells as in (A). Summary of results is shown on the right. Data are representative of three experiments of 5 mice per group. Statistical significance was assessed with Student’s t-test (B, C, F) with Holm-Sidak multiple comparison correction (D). **P < 0.01, ****P < 0.0001.

Having determined that high, persistent expression of CD39 is a feature of LCMV-specific CD8^+^ T cells during chronic LCMV infection, we next sought to further characterize the phenotype of CD39^+^ CD8^+^ T cells during Clone 13 infection. We analyzed CD39 expression in antigen-experienced, CD44^+^ CD8^+^ T cells and found that mice infected with Clone 13 developed a population of cells with particularly high expression of CD39 (CD39^high^). This population was entirely absent in mice infected with the acute LCMV Armstrong strain at d35 p.i., which only exhibited the presence of intermediate levels of CD39 staining (CD39^int^) (Fig 6A). Further characterization of the two sub-populations in Clone 13 infected mice revealed that the CD39^high^ cells showed more down-regulation of CD127 (Fig 6B) and higher expression of PD-1 (Fig 6C) than did the CD39^int^ population.

Because the highest levels of PD-1 are characteristic of terminally exhausted CD8^+^ T cells in chronic infection [12,33], we tested whether CD39^high^ T cells in chronic infection showed other phenotypic characteristics of terminal exhaustion. Analysis of expression of two additional co-inhibitory receptors, CD244 (2B4) and Lag3, showed that a significantly higher fraction of CD39^high^ cells co-expressed multiple receptors, consistent with terminal exhaustion. In contrast, CD39^int^ CD8^+^ T cells were generally negative for all three receptors analyzed (Fig 6D and 6E). We next examined the expression of the transcription factors T-bet and Eomes. We found that the CD39^high^ subset of CD8^+^ T cells was comprised primarily of Eomes^high^ T-bet^low^ terminally exhausted phenotype, while the CD39^int^ CD8^+^ T cells showed a comparable distribution of both (Fig 6F). Similarly, we found that in CD8^+^ T cells from subjects with either HCV or HIV infection, the CD39^+^ CD8^+^ T cell compartment contained a significantly higher ratio of Eomes^high^ T-bet^low^: Eomes^low^ T-bet^high^ relative to CD39^–^ CD8^+^ T cells (S4 Fig). Thus in both humans and mice with chronic viral infection, CD39^+^ CD8^+^ T cells show a phenotype consistent with previous descriptions of terminal exhaustion [9].

### CD39 correlates with reduced functionality in virus-specific CD8^+^ T cells in chronic infection

**Fig 7:**
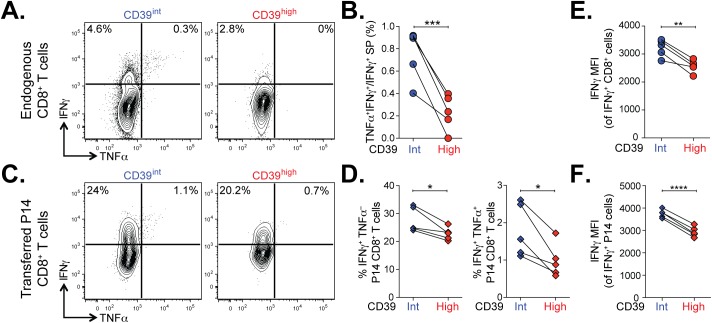
Terminally exhausted CD8^+^ T cells marked by high levels of CD39 are most impaired in their effector function. (**A**) Representative plots showing the production of IFN-γ and TNFα in CD39^int^ or CD39^high^ CD8^+^ T cells 36 days following LCMV Clone 13 infection. (**B**) Quantification of cells in (A) that produce both TNFα and IFN-γ relative to IFN-γ only. (**C, D**) Cytokine production by P14 cells (C) gated from an infection as in (A) and summary of IFN-γ and TNFα producing cells. (**E, F**) Mean fluorescence intensity (MFI) of IFN-γ in IFN-γ positive endogenous (E) and transferred P14 cells (F). Statistical significance was assessed with paired Student’s t-test. *P < 0.05, **P < 0.01, ***P < 0.001, ****P < 0.0001.

We next examined the functional properties of CD39^high^ and CD39^int^ CD8^+^ T cells from mice with chronic LCMV infection. Co-production of cytokines IFN-γ and TNFα is a feature of virus-specific T cells responding to acute infection and in the early stages of chronic infection but is progressively lost as exhaustion evolves [2]. To compare the functionality of CD39^high^ and CD39^int^ virus-specific CD8^+^ T cells, we isolated CD8^+^ T cells from mice with chronic infection at d35 post-infection and stained for IFN-γ and TNFα following in vitro stimulation with GP_33-41_ peptide. We found a significantly smaller fraction of antigen-specific coproduced IFN-γ and TNFα in CD39^high^ CD8^+^ T cells compared to CD39^int^ CD8^+^ T cells (Fig 7A and 7B).

To confirm this finding, we analyzed the function of transferred P14 CD8^+^ T cells in chronic infection. The P14 TCR transgene recognizes the GP_33-41_ peptide of LCMV presented on H-2D^b^. We found that both the frequency of IFN-γ-producing and IFN-γ/TNFα co-producing P14 T cells was significantly lower in CD39^high^ CD8^+^ T cells compared to CD39^int^ CD8^+^ T cells (Fig 7C and 7D). The defect in cytokine secretion was not only observed in terms of the frequency of cytokine-secreting cells, but also in the amount of cytokine detected per cell. Even among cells that did secrete IFN-γ, we found the MFI of expression to be significantly lower in CD39^high^ CD8^+^ T cells compared to CD39^int^ CD8^+^ T cells (Fig 7E and 7F). Thus high levels of CD39 expression demarcate a population of exhausted cells with the poorest function in chronic infection.

## Discussion

The state of CD8^+^ T cell exhaustion is characterized by widespread changes in gene expression relative to functional memory CD8^+^ T cells [5]. However, in humans, identification of specific T cell exhaustion markers that are not shared by more functional CD8^+^ T cell populations has been challenging [8]. We show that high-level expression of the ectonucleotidase CD39 is characteristic of CD8^+^ T cells specific for chronic viral infections in humans and mice, but is otherwise rare in the CD8^+^ T cell compartment of healthy donors. Persistent, high-level expression is also seen in the LCMV mouse model of chronic viral infection, suggesting that CD39 expression is a phenotypic marker of CD8^+^ T cell exhaustion. Moreover, within the exhausted population in the mouse model, CD39^high^ CD8^+^ T cells express the highest levels of PD-1, co-express multiple inhibitory receptors and have profoundly impaired function. We found that in both mice and humans, CD39 is expressed preferentially by CD8^+^ T cells that are T-bet^low^/Eomes^high^. These data suggest that CD39 expression by CD8^+^ T cells is a pathological finding and demarcates the population of CD8^+^ T cells previously identify as being terminally exhausted [9].

The fact that peripheral blood CD8^+^ T cells in humans can express CD39 is surprising. Previous data have shown that CD39 expression is restricted to CD4^+^ regulatory T cells, Th17 cells, and small populations of regulatory-like CD8^+^ T cells [14,21–23]. Indeed, we find that in the bulk population of CD8^+^ T cells in healthy donors only a small minority of CD8^+^ T cells expresses CD39. However, CD39 is abundantly expressed by virus-specific CD8^+^ T cells in two human chronic infections (HIV and HCV). This helps explain why CD39^+^ CD8^+^ T cells have not been appreciated in earlier studies that have focused on healthy individuals, and suggests that, in steady-state conditions, the expression of CD39 by CD8^+^ T cells is a pathological occurrence that is related to the development of T cell exhaustion. Whether the small fraction of CD8^+^ T cells expressing CD39 in healthy donors represents acutely activated CD8^+^ T cells, or those exhausted by asymptomatic chronic pathogens or inflammatory signals is an important question for future studies.

Several features of CD39-expressing CD8^+^ T cells suggest that CD39 is a diagnostically valuable marker of T cell exhaustion. First, in both human and mouse CD8^+^ T cells responding to chronic infection, CD39 is co-expressed with PD-1, an inhibitory receptor expressed by the majority of exhausted T cells [5,6]. Second, CD39 expression correlates with viral load in subjects with HIV and HCV infection suggesting that the conditions of high levels of inflammation and antigen load that lead to exhaustion also increase CD39 expression in the virus-specific pool of CD8^+^ T cells, as has been observed for PD-1 [3,34]. Third, gene signatures characteristic of exhausted mouse CD8^+^ T cells are enriched in CD39^+^ cells relative to CD39^–^ CD8^+^ T cells in subjects with HCV infection, underscoring the association between CD39 expression and T cell exhaustion. Finally, chronic LCMV infection in the mouse model increases CD39 expression by exhausted virus-specific CD8^+^ T cells, and elicits a population of CD39^high^ cells that are absent in functional memory cells. Previous studies show that CD39, like PD-1, is transiently up-regulated by acute T cell activation [14,35]. Additional studies will therefore be required to determine the extent to which T cell activation (rather than exhaustion *per se*) contributes to the up-regulation of CD39 and PD-1 in chronic infection. However, the strong association between CD39 expression and the hallmark phenotypic features of T cell exhaustion in humans and a mouse model suggests that it can serve as a valuable marker of the exhausted T cells state.

The expression of molecules, such as PD-1, that inhibit T cell function has been used to identify exhausted CD8^+^ T cells in several studies of human chronic infection and cancer [2]. However, there are important distinctions between the pattern of CD39 expression and that of inhibitory receptors. Many inhibitory receptors, such as PD-1 [3,8,36] and CD244 [37,38] are also expressed by a substantial fraction of CD8^+^ T cells in healthy donors that are not exhausted. In contrast, CD39 expression is found in only a very small minority of CD8^+^ T cells from healthy donors. This expression pattern suggests that CD39 expression, particularly in combination with PD-1, may be useful as a more specific phenotype of exhausted CD8^+^ T cells, at least in HCV and HIV infection. In addition, CD39 may provide a useful marker to isolate exhausted CD8^+^ T cells in settings such as tumor-specific responses where very few reagents are available to identify antigen-specific T cells. Importantly, while CD39 is rare in the CD8^+^ compartment in healthy donors, it is expressed by CD4^+^ Tregs–as is PD-1 –making it difficult to distinguish between exhausted CD4^+^ T cells and Tregs by CD39 expression alone.

Analysis of global expression profiles of CD39^+^ *versus* CD39^–^ CD8^+^ T cells in HCV-infected subjects showed that the CD39^+^ fraction was strongly enriched for genes related to proliferation. This may at first seem counterintuitive, given the functional defects that have been described in exhausted CD8^+^ T cells [2,5]. However, data from the mouse model of chronic infection suggest that, unlike memory CD8^+^ T cells, exhausted CD8^+^ T cells are dependent on continuous exposure to viral antigen to ensure their survival and undergo extensive cell division at a rate higher than that seen in physiological homeostatic proliferation of the memory CD8^+^ T cell pool [39]. Exhausted CD8^+^ T cells therefore have a paradoxical increase in their proliferation *in vivo* despite reduced proliferative potential *in vitro* [40], explaining the increased expression of proliferation-associated genes in CD39^+^ CD8^+^ T cells in HCV infection and in mouse exhausted CD8^+^ T cells [9,41].

Recent studies of exhausted CD8^+^ T cells have revealed that two distinct states of virus-specific CD8^+^ T cells exist in chronically infected mice and humans [9,10]. Differential expression of the T-box transcription factors T-bet and Eomes characterize two populations, which form a progenitor-progeny relationship. T-bet^high^ cells display low intrinsic turnover but are capable of proliferation in response to persisting antigen, giving rise to Eomes^high^ terminal progeny. In contrast, Eomes^high^ CD8^+^ T cells responding to chronic infection had reduced capacity to undergo additional proliferation *in vivo*. The T-bet^low^ /Eomes^high^ exhausted subset of CD8^+^ T cells correspond to the PD-1 bright population that has also been shown to be unresponsive to PD-1:PD-L1 blockade. These data suggest that the differential expression of these transcription factors identifies subpopulations of exhausted CD8^+^ T cells with fundamentally different fates and functional profiles. Our data show that in the LCMV mouse model of chronic infection and in HIV infection, the CD39^high^ subset of CD8^+^ T cells demarcates T-bet^low^ /Eomes^high^ cells. Consistent with this, CD39^+^ CD8^+^ T cells in the mouse model express the highest levels of PD-1, co-express multiple inhibitory receptors and show marked functional defects. These findings suggest that CD39 may be a marker not only of the exhausted state, but specifically of the most terminally exhausted cells, at least in the mouse model. Additional studies of the fate of transferred CD39^+^ vs. CD39^–^ exhausted CD8^+^ T cells in the mouse model, and broader surveys of CD39 expression in human chronic infections will be required to determine whether this marker can be used as a surrogate for terminal exhaustion. However, the strong association between CD39 expression and the key features of terminal exhaustion suggests that it may prove a useful marker to help distinguish between "reversible" and "irreversible" T cell exhaustion. Moroever, the fact that isolating CD39^+^ cells does not require intracellular staining (as is required for T-bet and Eomes) makes this marker useful for studying the function of this terminally exhausted cells *ex vivo*.

The fact that CD39 is expressed by a slightly larger fraction of HCV-specific CD8^+^ T cells than HIV-specific CD8^+^ T cells may be related to differences in the timing of blood sampling during the course of infection, or may be due to differences in the extent of antigen-load and inflammation in the two infections. Alternatively, it may be consistent with a model in which HCV-specific CD8^+^ T cells are in a more “terminal” state of exhaustion than CD8^+^ T cells specific for HIV. This possibility is supported by profound loss of HCV-specific CD8^+^ T cells over the course of chronic infection [42] that is not seen in the HIV-specific CD8^+^ T cell pool, consistent with the clonal deletion seen in mouse models of extreme CD8^+^ T cell exhaustion [43,44]

It is tempting to speculate that expression of CD39 contributes to the dysfunction of exhausted T cells [45]. For instance, the expression of CD39 might enable CD8^+^ T cells to provide negative regulation in an autocrine or juxtacrine fashion via adenosine [18–20] in the same manner as Tregs [15,35]. The fact that CD39 requires both a substrate (ATP) and a downstream enzyme (CD73) to generate adenosine could provide a mechanism to ensure that this negative signaling occurred only in certain contexts such as in inflamed, damaged tissues where the extracellular concentrations of ATP are high and CD73-expressing cells are present [46]. Moreover, CD39-expressing CD8^+^ T cells may contribute to the general inhibitory milieu by contributing to the inhibition of activated T cells that express the adenosine receptor but are not yet exhausted. It will therefore be important to determine whether inhibition of CD39 activity could provide an additional therapeutic strategy to rescue the function of exhausted T cells.

## Materials and Methods

### Human Subjects

Healthy human donors were recruited at the Kraft family Blood Donor Center, Dana-Farber Cancer Institute. All human subjects with HCV infection were recruited at the Gastrointestinal Unit and the Department of Surgery of the Massachusetts General Hospital (Boston, MA) (S1 Table).

Individuals with chronic HCV infection (n = 82) were defined by positive anti-HCV antibody and detectable viral load. Patients with spontaneous clearance of HCV, termed resolvers (n = 30), were defined by positive anti-HCV antibody but an undetectable viral load for at least 6 months. The estimated time of infection was calculated either using the exposure date or the time of onset of symptoms and peak ALT (which are assumed to be 7 weeks post infection). All HCV patients were treatment naive and studied at 5.9 and 219.7 weeks post infection. HCV RNA levels were determined using the VERSANT HCV RNA 3.0 (bDNA 3.0) assay (Bayer Diagnostics).

All HIV infected subjects (n = 40) were recruited at the Ragon Institute at the Massachusetts General Hospital (Boston, USA) or the Peter Medawar Building for Pathogen Research (Oxford, UK) (S2 Table). HIV controllers included elite controllers (n = 5) defined as having HIV RNA below the level of detection (<75 viral copies per ml) and viremic controllers (n = 7) with HIV RNA levels < 2,000 viral copies per ml. HIV chronic progressors (n = 28) were defined as having > 2,000 viral copies per ml. All subjects were off therapy. Viral load during chronic infection was measured using the Roche Amplicor version 1.5 assay.

### MHC Class I Tetramers

Major histocompatibility complex (MHC) class I HIV Gag-specific tetramers were produced as previously described [47] or obtained from Proimmune. CMV- and EBV-specific MHC class I dextramers conjugated with FITC and APC were purchased from Immudex. Mouse MHC class I tetramers of H-2D^b^ complexed with LCMV GP_276-286_ were produced as previously described [48,49]. Biotinylated complexes were tetramerized using allophycocyanin-conjugated streptavidin (Molecular Probes). The complete list of multimers can be found in supplemental materials (S3 Table).

### Antibodies and flow cytometry

The following anti-human (hu) and anti-mouse (m) fluorochrome-conjugated antibodies were used for flow cytometry: huCD8α (RPA-T8), huCD4 (OKT4), huCD3 (OKT3), huCD39 (A1), huPD-1 (EG12.2H7), huCD25 (BC96), huCCR7 (G043H7), huCD45RA (HI100), huT-bet (4B10), mCD8α (53–6.7), mCD4 (GK1.5), mCD3 (145-2C11), mCD244.2 (m2B4 (B6)458.1), mPD-1 (RMP1-30), mLag3 (C9B7W), mCD44 (IM7), mCD127 (A7R34), mTNFα (MP6XT22) (all from Biolegend), mT-bet (04–46; BD Pharmingen), mCD39 (24DMS1), mIFN-γ (XMG1.2), huEomes (WD1928) and mEomes (Dan11mag) (eBioscience). Intracellular staining was performed following surface staining and fixed and permeabilized using the FoxP3/Transcription Factor Staining Buffer Set (eBioscience). Cells were sorted by BD FACS ARIA II and all other analyses were performed on BD LSR II and BD LSR Fortessa flow cytometers equipped with FACSDiva v6.1. Gates were set using Full Minus One (FMO) controls. Data were analyzed using FlowJo software v9.8 (Treestar).

For intracellular cytokine analysis of mouse T cells, 2x10^6^ splenocytes were cultured in the presence of GP_33-41_ peptide (0.2 μg/ml) (sequence KAVYNFATM), brefeldin A (BD), and monensin (BD) for 4.5 hours at 37°C. Following staining for surface antigens, cells were permeabilized and stained for intracellular cytokines with the Cytofix/Cytoperm kit according to manufacturer's instructions (BD Biosciences).

### Mice and infections

Wild-type C57BL/6J mice were purchased from The Jackson Laboratory. Female mice (6–8 weeks old) were infected with 2 x 10^5^ plaque forming units (p.f.u.) of LCMV-Armstrong intraperitoneally or 4 x 10^6^ p.f.u. of LCMV-Clone 13 intravenously and analyzed at indicated time points by homogenizing the spleen into a single-cell suspension, Ammonium-Chloride-Potassium lysis of red blood cells, followed by antibody staining. For experiments involving P14 cell transfers, Ly5.1+ P14s were isolated from peripheral blood, and 500 P14 cells were transferred i.v. into 5–6 week old wild-type female mice one day prior to infection. Viruses were propagated as described previously [48–50].

### HPLC analysis of ATP levels

The concentration of ATP hydrolyzed by CD8^+^ T cells from subjects with HCV infection (n = 6) was assessed by high performance liquid chromatography (HPLC) as previously described [51]. Briefly, 10,000 CD39^+^ CD8^+^ T cells were sorted and placed on ice to minimize ATP production by cells. 20 μM of ATP was added and incubated for 1 h at 37°C in 5% CO_2_ to allow for cellular activity to increase and CD39-mediated ATP hydrolysis to occur. Samples were then placed in an ice bath for 10 min to halt enzymatic activity, collected, and centrifuged for 10 min at 380 x g and 0°C. Cells were discarded and supernatant centrifuged again to remove remaining cells (2350 x g, 5 min, 0°C). The resulting RPMI samples (160 μl) were treated with 10 μl of an 8 M perchloric acid solution (Sigma-Aldrich) and centrifuged at 15,900 x g for 10 min at 0°C to precipitate proteins. In order to neutralize the pH of the resulting solutions and to remove lipids, supernatants (80 μl) were treated with 4 M K_2_HPO_4_ (8 μl) and tri-N-octylamine (50 μl). These samples were mixed with 50 μl of 1,1,2-trichloro-trifluoroethane and centrifuged (15,900 x g, 10 min, 0°C) and this last lipid extraction step was repeated once. The resulting supernatants were subjected to the following procedure to generate fluorescent etheno-adenine products: 150 μl supernatant (or nucleotide standard solution) was incubated at 72°C for 30 min with 250 mM Na_2_HPO_4_ (20 μl) and 1 M chloroacetaldehyde (30 μl; Sigma-Aldrich) in a final reaction volume of 200 μl, resulting in the formation of 1,N6-etheno derivatives as previously described [51]. Samples were placed on ice, alkalinized with 0.5 M NH_4_HCO_3_ (50 μl), filtered with a 1 ml syringe and 0.45 μM filter and analyzed using a Waters HPLC system and Supelcosil 3 μM LC-18T reverse phase column (Sigma), consisting of a gradient system described previously, a Waters autosampler, and a Waters 474 fluorescence detector [52]. Empower2 software was used for the analysis of data and all samples were compared with water and ATP standard controls as well as a sample with no cells to determine background degradation of ATP.

### Microarray data acquisition

CD8^+^ T cells from subjects with HCV infection were sorted and pelleted and re-suspended in TRIzol (Invitrogen). RNA extraction was performed using the RNAdvance Tissue Isolation kit (Agencourt). Concentrations of total RNA were determined with a Nanodrop spectrophotometer or Ribogreen RNA quantification kits (Molecular Probes/Invitrogen). RNA purity was determined by Bioanalyzer 2100 traces (Agilent Technologies). Total RNA was amplified with the WT-Ovation Pico RNA Amplification system (NuGEN) according to the manufacturer's instructions. After fragmentation and biotinylation, cDNA was hybridized to HG-U133A 2.0 microarrays (Affymetrix). Data have been deposited in Gene Expression Omnibus with accession code GSE72752.

### Statistics

Prior to analysis, microarray data were pre-processed and normalized using robust multichip averaging, as previously described [53]. Differentially gene expression and consensus clustering [54] were performed using Gene-E software (www.broadinstitute.org/cancer/software/GENE-E/), and gene set enrichment analysis was performed as described previously using gene sets from MSigDB [55] or published resources [29,32].

Consensus hierarchical clustering was performed using the top 10% of genes that varied across the dataset, without reference to sample identity. Consensus cluster assesses the “stability” of the clusters discovered using unbiased methods such as hierarchical clustering i.e. the robustness of the putative clusters to sampling variability. The basic assumption is that if the data represent a sample of items drawn from distinct sub-populations, a different sample drawn from the same sub-populations, would result in cluster composition and number should not be radically different. Therefore, the more the attained clusters are robust to sampling variability, the greater the likelihood that the observed clusters represent real structure. The result of consensus clustering is a matrix that shows, for each pair of samples, the proportion of clustering runs on sub-sampled data in which those two items cluster together (shown on a scale of 0 to 1).

Enrichment Map analysis of GSEA results was performed as described [56]. The gene signature of exhaustion was generated by identifying the top 200 genes upregulated in CD8^+^ T cells responding to chronic vs. acute LCMV infection in microarray data from a previously published study [29].

### Ethics statement

All human subjects were recruited with recruited with written informed consent in accordance with Dana-Farber Cancer Institute IRB approval DFCI 00–159, Partners IRB approvals 2010P002121, 2010P002463, 1999P004983, and Oxford Research Ethics Committee approval 06/Q1604/12. The mouse work was performed under a protocol 01214 approved by the HMA Institutional Animal Care and Use Committee (IACUC), in strict accordance with the recommendations in the Guide for the care and use of Laboratory Animals of the National Institutes of Health. The Harvard Medical School animal management program is accredited by the Association for the Assessment and Accreditation of Laboratory Animal Care International (AAALAC).

## Supporting Information

S1 Fig CD39 is expressed by few CD8^+^ T cells in health donors. Fraction of CD39^+^ cells in naïve CD8^+^ T and central memory (CM), effector memory (EM) and effector memory RA^+^ (EMRA) subpopulations of CD8^+^ T cells based on CD45RA and CCR7 staining from 18 healthy human donors. Error bars represent SEM. Statistical significance was assessed by Friedman test. **P <0.01, ***P <0.001.
(TIF)

S2 Fig CD39 and PD-1 co-expression in HCV and HIV. (A, B) Fraction of HCV-specific (A) and HIV-specific (B) CD8^+^ T cells expressing PD-1, CD39, or both in patients with persistent high viral load (black) or patients controlling the disease (grey). Correlation of the fraction of PD-1 and CD39 double positive virus specific CD8^+^ T cells with the viral load in the blood in HCV (C) and HIV (D) infected patients. Statistical significance was assessed by Mann-Whitney test with Bonferroni correction (A, B). *P <0.05. Correlation was assessed by Pearson correlation coefficient (C, D). MFI; mean fluorescence intensity.
(TIF)

S3 Fig Cell sorting strategy for microarray analysis. Gating strategy for CD39^+^ and CD39^–^ live non-naive CD8^+^ T cells from HCV-infected patients.
(TIF)

S4 Fig Comparison of T-bet and Eomes expression by CD39^+^ and CD39^–^ CD8^+^ T cells in patients with chronic viral infection. (**A, D**) Expression of CD39 in CD8^+^ T cells in patients infected with HCV (A) and HIV (D). (**B, E**) Expression of transcription factors T-bet and Eomes on CD39^–^ and CD39^+^ populations identified in (A) and (D). (**C, F**) Summary of the ratio of terminally exhausted Eomes^high^/T-bet^low^ CD8^+^ T cells in CD39^–^ and CD39^+^ subsets in HCV (C) and HIV (F) infection. Statistical significance was assessed with paired Student’s t-test. *P < 0.05, ***P < 0.001.
(TIF)

S1 Table Clinical characteristics of the subjects with HCV infection. (XLSX)

S2 Table Clinical characteristics of the subjects with HIV infection. (XLSX)

S3 Table The complete list of MHC-peptide multimers used in the study. (XLSX)

S4 Table List of genes differentially expressed in CD39^+^ vs CD39^–^ CD8^+^ T cells in HCV infected subjects (FDR<0.15). (XLSX)
