# Supplementary figures and images for: CD39 Expression Identifies Terminally Exhausted CD8+ T Cells
Source: PLoS Pathog. 2015 Oct 20;11(10):e1005177. doi: 10.1371/journal.ppat.1005177 (PMC4618999; doi:10.1371/journal.ppat.1005177)

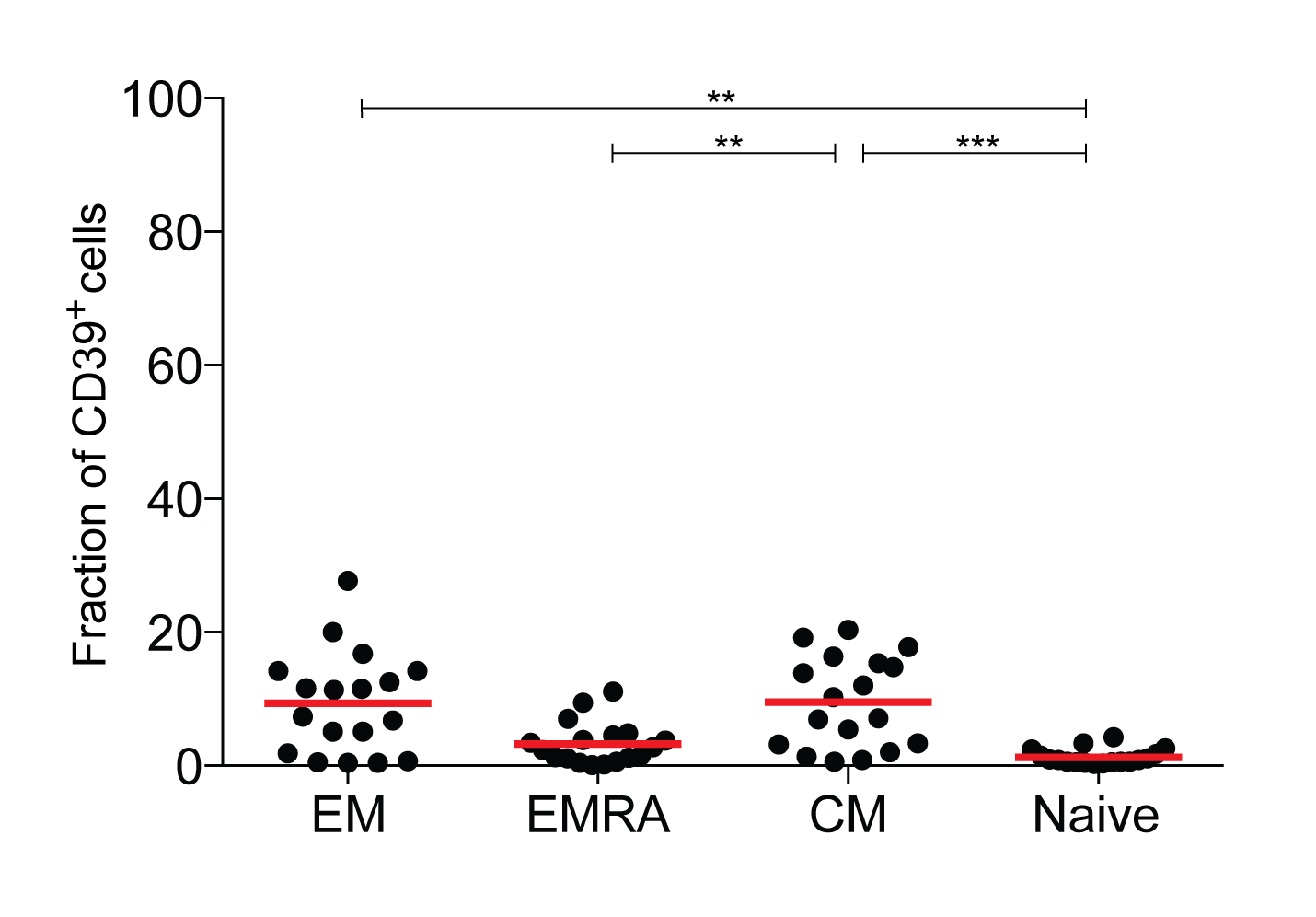

Supplement: S1 Fig — Fraction of CD39+ cells in naïve CD8+ T and central memory (CM), effector memory (EM) and effector memory RA+ (EMRA) subpopulations of CD8+ T cells based on CD45RA and CCR7 staining from 18 healthy human donors. Error bars represent SEM. Statistical significance was assessed by Friedman test. **P <0.01, ***P <0.001. (TIF) [file ppat.1005177.s001.tif]

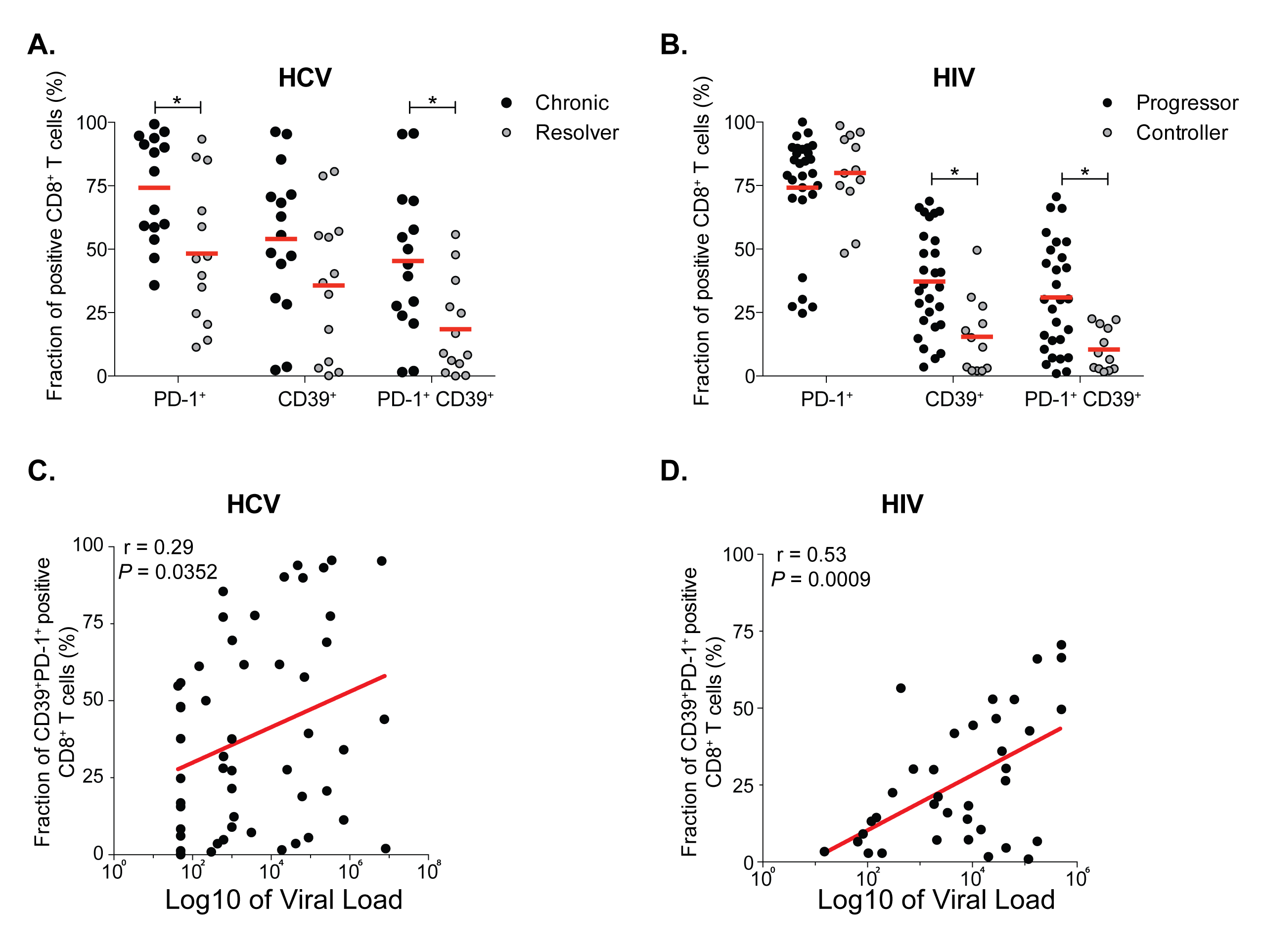

Supplement: S2 Fig — (A, B) Fraction of HCV-specific (A) and HIV-specific (B) CD8+ T cells expressing PD-1, CD39, or both in patients with persistent high viral load (black) or patients controlling the disease (grey). Correlation of the fraction of PD-1 and CD39 double positive virus specific CD8+ T cells with the viral load in the blood in HCV (C) and HIV (D) infected patients. Statistical significance was assessed by Mann-Whitney test with Bonferroni correction (A, B). *P <0.05. Correlation was assessed by Pearson correlation coefficient (C, D). MFI; mean fluorescence intensity. (TIF) [file ppat.1005177.s002.tif]

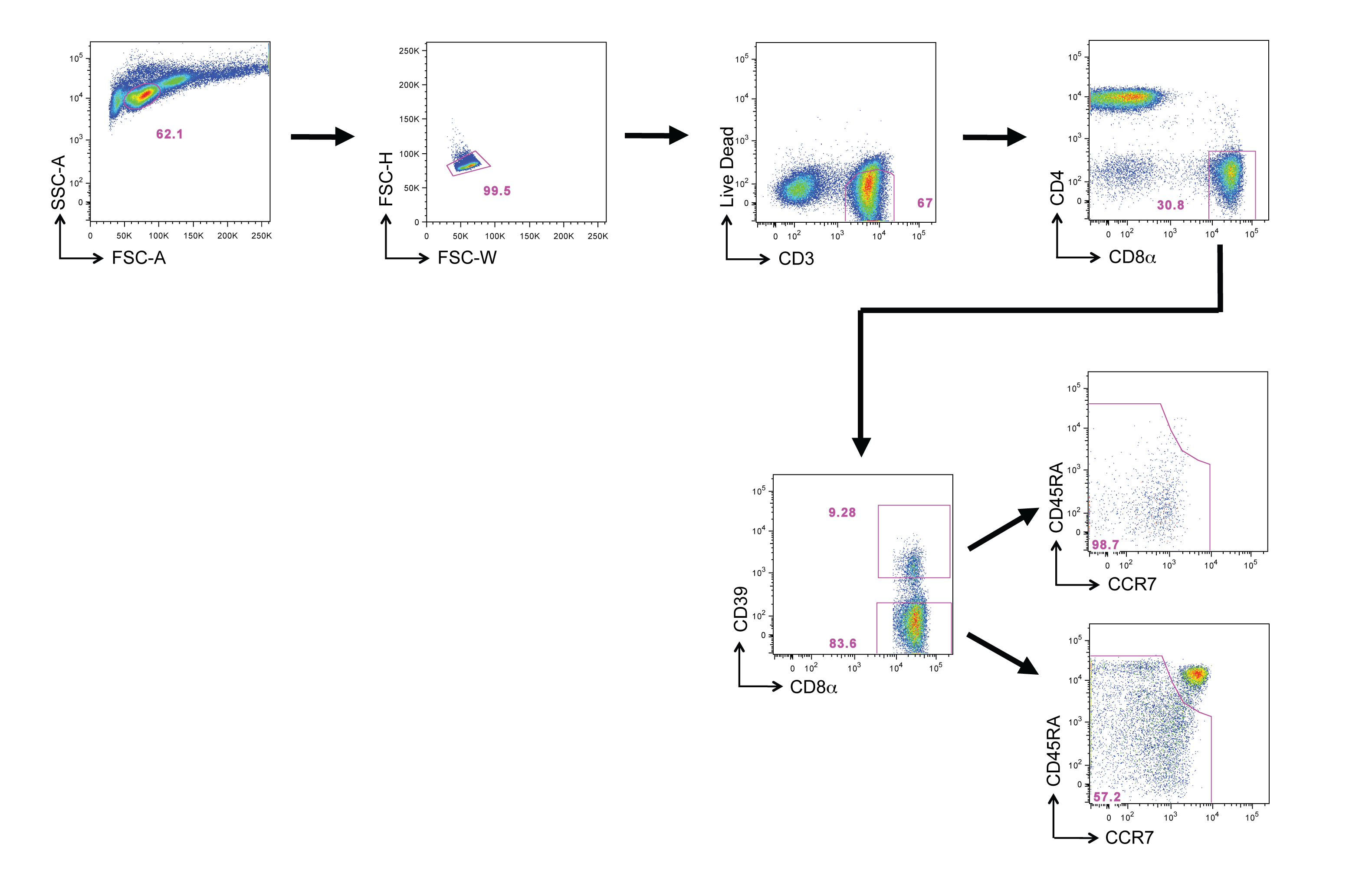

Supplement: S3 Fig — Gating strategy for CD39+ and CD39– live non-naive CD8+ T cells from HCV-infected patients. (TIF) [file ppat.1005177.s003.tif]

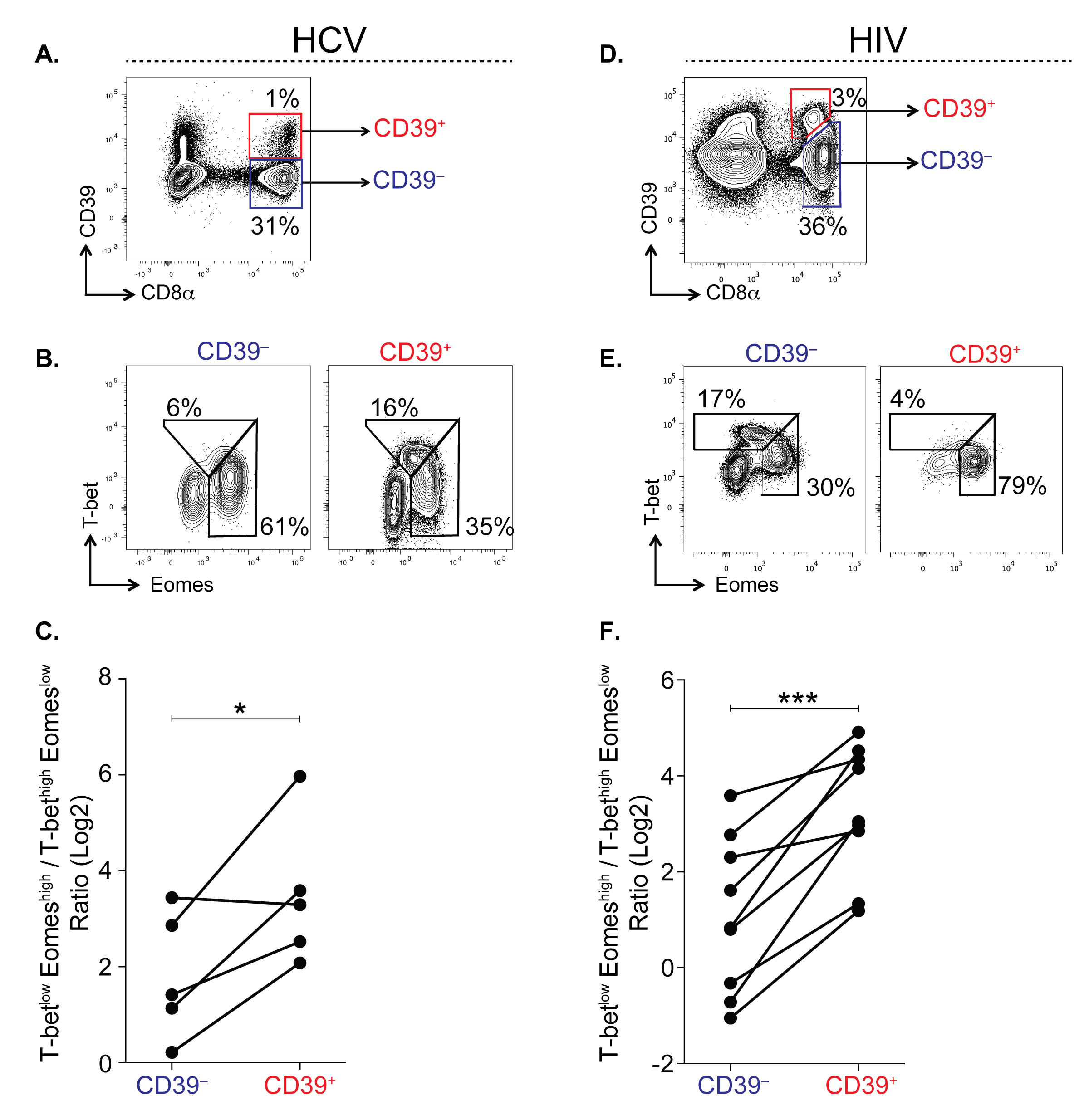

Supplement: S4 Fig — (A, D) Expression of CD39 in CD8+ T cells in patients infected with HCV (A) and HIV (D). (B, E) Expression of transcription factors T-bet and Eomes on CD39– and CD39+ populations identified in (A) and (D). (C, F) Summary of the ratio of terminally exhausted Eomeshigh/T-betlow CD8+ T cells in CD39– and CD39+ subsets in HCV (C) and HIV (F) infection. Statistical significance was assessed with paired Student’s t-test. *P < 0.05, ***P < 0.001. (TIF) [file ppat.1005177.s004.tif]
